# Polyphenol–Inorganic Sulfate Complex-Enriched Straightening Shampoo for Reinforcing and Restoring Reduced Hair Integrity

**DOI:** 10.3390/biomimetics10030132

**Published:** 2025-02-22

**Authors:** Tae Min Kim, Heung Jin Bae, Sung Young Park

**Affiliations:** 1 Department of IT and Energy Convergence (BK21 FOUR), Korea National University of Transportation, Chungju 27469, Republic of Korea; ktm1120@a.ut.ac.kr; 2 MODAMODA Corporation, Ltd., Songpa-gu, Seoul 05546, Republic of Korea; 3 Department of Chemical and Biological Engineering, Korea National University of Transportation, Chungju 27469, Republic of Korea

**Keywords:** polyphenol, sodium sulfate, disulfide bond, hair straightening, haircare, shampoo

## Abstract

Conventional hair-straightening methods that use chemical treatments to break disulfide bonds cause severe damage to the hair shaft, leading to weakened hair that is prone to reverting to its curly form in high humidity. Therefore, a unique haircare coating technology is required to protect hair integrity and provide a long-lasting straightening effect. Herein, we designed a hair-straightening technology by integrating a nature-inspired polyphenol–inorganic sulfate (PIS) redox agent into formulated shampoo, which achieves a desirable straightening effect through sulfate-induced disulfide breakage while preserving hair integrity through a polyphenol-reinforced structure. The interaction between polyphenols and residual thiols from the straightening process maintained a long-lasting straight hair structure and hair strength. Ellman’s assay showed a lower free thiol content from reductant-induced damaged keratin in PIS shampoo-treated hair than in sulfate-treated hair as the polyphenol–thiol bond was formed through the Michael addition reaction, thereby restoring the natural structure of the hair and enhancing its mechanical properties. Owing to the polyphenol coating, PIS shampoo-treated hair exhibited an antistatic effect and high hydrophobicity, indicating healthy hair. Furthermore, the polyphenol coating effectively scavenged radical oxygen species (ROS) in the hair, thereby improving damage protection. Thus, PIS shampoo offers an alternative approach for effective hair straightening.

## 1. Introduction

The significant development of the internet and social media over the last few decades has encouraged an increased focus on physical appearance, as an increasing number of individuals express themselves through social media and value feedback from others [1,2]. Consequently, numerous individuals have invested more time and money in skincare, hair treatment, and fashion, thereby driving growth in these industries [3,4]. Particularly for hair treatment, various hair products have emerged to support the high demand for obtaining a desirable hair look despite their potential side effects or hair damage [5,6]. Hair styling is an important factor that plays a significant role in the external image of an individual. As interest in hair styling grows, the demand for straight hair, driven by social stereotypes of attractiveness, its versatility in styling, and ease of daily maintenance, also increases [7,8]. Human hair characteristics vary depending on genetics, and individuals with curly or semi-curly hair often face difficulties in styling their hair because these hair types are difficult to manage and are prone to frizz [9,10,11]. High humidity and temperature, particularly in summer, can cause styled hair to easily become loose, limp, or frizzy [12,13,14]. To address these challenges, hair-straightening treatments have become popular (market size in 2024 = USD 633.3 million; projected to increase to USD 854.2 million in 2033), which aim to straighten naturally curly, semi-curly, or artificially modified hair by eliminating the curves and creating smooth and straight hair [15]. To this end, these products can temporarily or semi-permanently change the shape of the hair by changing the hydrogen bonds or disulfide bonds of the hair that play a crucial factor in the shape and structure of the hair [16,17,18,19].

A common and simple hair-straightening method involves using devices such as dryers or irons. During the drying process, moisture evaporates from the hair, and hydrogen bonds form between protein peptide chains, temporarily straightening the hair [20,21]. However, these bonds are easily broken when moisture re-penetrates the hair owing to humidity, thus causing it to revert to its original state [22,23]. Chemical-based hair-straightening methods, such as using inorganic sulfate compounds as reducing agents, are also commonly used [24,25,26]. Reducing agents break disulfide bonds within the hair, altering its structure and loosening curls, resulting in a neater appearance [27,28]. Hair treated using this approach tends to maintain a straight structure longer than those treated with a drying process. However, exposure to a reducing agent often damages the entire hair shaft, weakening hair strength. In addition, the resulting straight hair remains prone to becoming frizzy in high humidity [29,30]. To address this issue, polyphenols, which are naturally occurring secondary metabolites, have been considered owing to their antioxidizing and anti-inflammatory effects, along with notable adhesive properties, such as those in mussels [31,32,33]. The addition of polyphenol compounds, such as those from the commercially available apple extract and green tea leaf extract (GTLE) containing epigallocatechin gallate (EGCG) and quercetin, to haircare products as coating materials inspired by mussel adhesive has been shown to promote structural reinforcement that increases the mechanical properties of hair and protects it against free radicals that induce hair damage [34,35,36,37,38]. Combining polyphenols with a reducing agent for hair straightening may produce synergistic effects, thereby resulting in long-lasting straight hair with improved mechanical strength and smoothness [39,40].

In the present study, we developed a unique hair-straightening technology that integrated a polyphenol–inorganic sulfate (PIS) redox agent into a formulated shampoo with the aim of achieving a safe, healthy, and effective straightening effect through sulfate-mediated disulfide bond cleavage, while maintaining hair integrity, which was reinforced by multiple interactions with polyphenols. The thiol group that is present in damaged hair following reduction by inorganic sulfate interacted with polyphenols through the Michael addition reaction, which repaired damage, strengthened hair integrity, and preserved the straight form after application of the PIS shampoo. Owing to the presence of polyphenols coating the hair surface, the hydrophobicity of hair was retained, thereby preventing the penetration of moisture that can disrupt the straight structure of hair. In addition, an antistatic effect was achieved because of the electroconductivity of polyphenols. Furthermore, the PIS shampoo showed reactive oxygen species (ROS) scavenging capability, which is necessary for preventing hair damage. Based on these extensive experiments, we determined that PIS shampoo is a potentially useful haircare technology for multifunctional hair-straightening applications.

## 2. Materials and Methods

### 2.1. Materials and Characterization

GTLE was obtained from Gaia Herbs (Brevard, NC, USA). Dopamine hydrochloride (DOPA), salicylic acid, H_2_O_2_, 2,2-diphenyl-1-picrylhydrazyl (DPPH), FeSO_4_, NaOH, 5,5′-dithio-bis-(2-nitrobenzoic acid) (DTNB), phosphate-buffered saline (PBS), trizma base solution (TBS), and tris-HCl were purchased from Sigma-Aldrich (Seoul, Republic of Korea). Sodium sulfate (SS) was obtained from Samchun Chemicals (Pyeongtaek, Republic of Korea). The shampoo base (MODA Zero Damage Shampoo) was provided by MODAMODA Corporation, Ltd. (Seoul, Republic of Korea). Silicon (Si) wafer (P-type) was purchased from Silicon Technology Corporation (Daejeon, Republic of Korea). Detroit 551 human skin fibroblast cells were obtained from the Korea Cell Line Bank (Seoul, Republic of Korea). Cell staining dyes calcein acetoxymethyl (calcein-AM; green, live) and propidium iodide (PI; red, dead) for live and dead staining assays were purchased from Thermo Fisher Scientific, Incheon, Republic of Korea. For the 3-(4,5-dimethyl-2-thiazolyl)-2,5-diphenyl-2H-tetrazolium bromide (MTT) assay, MTT was purchased from Sigma-Aldrich (Seoul, Republic of Korea). For the hair-straightening experiment, we used commercially available hair models purchased from Beauty Damoa (50% gray, Ansan, Republic of Korea) and Suyu General Distribution (Yeosinhair, pale beige, Wella T15, Seoul, Republic of Korea).

Fourier-transform infrared spectroscopy (FTIR) analysis was conducted using a Thermo Scientific^TM^ Nicolet^TM^ iS10 spectrometer (Thermo Fisher Scientific, Waltham, MA, USA). The ultraviolet–visible spectra (UV-Vis) were recorded using an Optizen 2020UV spectrometer (Mecasys, Daejeon, Republic of Korea). Photoluminescent spectra (PL) were measured using an L550B PL spectrometer (Perkin Elmer, Waltham, MA, USA). Thermal properties were determined through thermal gravimetric analysis (TGA) and differential scanning calorimetry using a DSC2910 differential thermal analyzer (TA Instruments, New Castle, DE, USA) (heating rate = 10 °C/min, N_2_ atmosphere). Scanning electron microscopy (SEM) was performed using a JSM-6700F microscope (JEOL, Musashino, Akishima, Tokyo, Japan). Surface roughness was determined using an XE-100 atomic force microscope (PSIA, Sungnam, Republic of Korea). Tensile strength was measured using a SurTA 1A Universal Testing Machine (Chemilab Co., Seoul, Republic of Korea). Confocal laser scanning microscopy (CLSM) images were captured using an ECLIPSE Ti2-E confocal microscope (Nikon, Tokyo, Japan). A multimode microplate reader (FilterMax F3, Molecular Devices, San Jose, CA, USA) was used for cell viability measurement using MTT assay.

### 2.2. Preparation of PIS Shampoo

PIS shampoo was prepared by mixing GTLE (0.45%), DOPA (0.06%), and SS (1.41%) with double-distilled water (DDW; 8.92%) in a vial. The mixture was stirred for 5 min at 60 °C under an N_2_ atmosphere, followed by continuous stirring at 25 °C for 12 h. Subsequently, the shampoo base (89.16%) was added to the PIS solution, and the mixture was stirred at 50 °C for 5 min. The resulting PIS shampoo was labeled S-D, whereas control shampoos (without one or more of the precursor components) were labeled S-A (shampoo only), S-B (shampoo + SS), and S-C (shampoo + GTLE–DOPA) (Supplementary Table S1). For nanoparticle characterization, the PIS mixture (without the shampoo base) was freeze-dried to obtain PIS nanoparticles (labeled D), whereas the control groups of nanoparticles were labeled A (DDW only), B (SS only), and C (GTLE–DOPA only).

### 2.3. Application of PIS Shampoo to Hair

Pretreatment was performed before applying the PIS shampoo to the hair by washing the hair with running water for 10 min. The PIS shampoo was then gently and evenly rubbed onto the hair; this process was repeated 30 times. The samples were placed in plastic dishes at room temperature for 30 min, then rinsed with running water and stored at room temperature for 1 h before drying.

### 2.4. Investigation of the Conductivity and Surface Coatability of PIS Nanoparticles

To evaluate the conductivity and surface coating ability of PIS nanoparticles, a PIS-coated surface was prepared on a silicon (Si) wafer with an area of 1 × 1 cm. The PIS nanoparticles were dissolved in TBS at pH 8.5 to a concentration of 5 mg/mL. The Si wafer was soaked in the PIS solution for 12 h and dried prior to measurement. The resistance change in the PIS-coated surfaces was measured using a source meter (Keithley 2450) at a bias voltage of 1 V using the two-electrode technique. For the surface coatability, the static contact angle was determined using a KRUSS DSA100 instrument and analyzed using the Cecil drop method. Each measurement was performed in triplicate (*n* = 3).

### 2.5. Assessment of Antistatic Effect of PIS Shampoo-Treated Hair

The antistatic effect of PIS shampoo on hair was evaluated using a simple electrostatic test with an air-filled balloon rubbed with cotton fabric. The electrostatic phenomenon was assessed when the balloon was placed close to the treated hair.

### 2.6. Measurement of Mechanical Properties (Tensile Strength) of PIS Shampoo-Treated Hair

To assess the mechanical properties of PIS shampoo-treated hair, the tensile strength of the treated hair was measured by fixing a hair strand in a UTM grip (stationary and dynamic grippers) at a strain rate of 10 mm/s (*n* = 3).

### 2.7. Assessment of ROS Scavenging Activity

The ROS scavenging activity of the PIS shampoo was assessed using DPPH and superoxide (O_2_^•−^) scavenging assays [41,42]. DPPH solution (0.1 mM, 3 mL) was added to the PIS solution (3 mL) and incubated at 37 °C for 30 min. The absorbance of the solution was measured at 517 nm using a UV-Vis spectrophotometer. For the O_2_^•−^ scavenging assay, the PIS solution (0.5 mL) was mixed with tris-HCl buffer (pH 8.2, 3 mL) and pyrogallol solution (0.05 M, 0.8 mL) and allowed to react at 25 °C for 5 min. Finally, HCl (8 M, 1 mL) was added and the absorbance was measured at 325 nm using a UV-Vis spectrophotometer. The ROS scavenging activity (DPPH and O_2_^•−^ assays) was calculated using the following equation:

ROS scavenging performance (%) = ((A_std_ − A_sam_)/A_std_) × 100%

where A_std_ is the absorbance of the standard solution and A_sam_ is the absorbance of the sample.

### 2.8. In Vitro Experiments and Cellular ROS Assay

Human skin fibroblast (Detroit 551) cells were cultured in high-glucose Dulbecco’s Modified Eagle Medium (DMEM) supplemented with 10% fetal bovine serum (FBS), 100 µg/mL streptomycin, and 100 units/mL penicillin. Cells (10^5^ cells/well) were then incubated with PIS solution (in PBS) for 24 h at 37 °C in a humidified incubator (5% CO_2_ atmosphere). For live and dead staining assay using CLSM, the cells were stained with calcein-AM and PI before being investigated using a confocal microscope (magnification: 40×). For cell viability analysis using the MTT assay, the MTT solution was added after the removal of the media. The cell viability was determined using a microplate reader with an absorbance wavelength of 570 nm. The intracellular ROS levels were determined using a #K936-100 ROS detection kit (amsbio, Cambridge, MA, USA), according to the manufacturer’s instructions.

### 2.9. Ellman’s Assay

DTNB (4 mg) was dissolved in 1 mL of sodium dodecyl sulfate (SDS) aqueous solution (2%) and then 90 μL of the resulting solution was diluted in 4.91 mL of SDS solution (2%) to prepare a DTNB solution. We immersed the PIS shampoo-treated hair in the DTNB solution and incubated it for 15 min at room temperature. The TNB concentration (formed by the interaction between DTNB and residual thiol from hair) was then measured and calculated by comparing the absorbance peaks at 412 nm based on the linear calibration curve.

### 2.10. UV Protection Assessment of PIS Shampoo-Treated Hair

UV protection assessment was performed by exposing hair samples (untreated hair/control and PIS shampoo-treated hair) to UV light at 302 nm for 1 h. Subsequently, the UV-irradiated hair samples were subjected to an Ellman’s assay analysis using the previously described method. The results are shown as a TNB concentration at an absorbance peak of 412 nm.

## 3. Results and Discussion

### 3.1. Design of PIS Shampoo for Hair-Straightening Treatment

**Figure 1:**
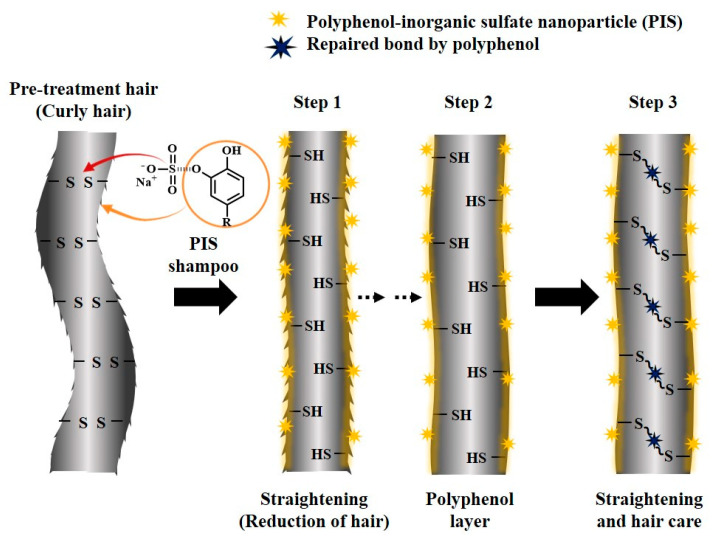
Schematic illustration of the effect of polyphenol–inorganic sulfate (PIS) shampoo on hair-straightening and integrity maintenance.

We developed PIS shampoo as a novel haircare technology for multifunctional hair-straightening and protection. It was fabricated by carefully mixing GTLE, DOPA, and SS (PIS nanoparticles) into a shampoo base (MODA Zero Damage Shampoo) to achieve synergistic hair straightening through SS and hair integrity maintenance through the GTLE–DOPA complex (polyphenols) (Supplementary Scheme S1). Polyphenol reacts with the sulfate of SS to form PIS nanoparticles that further play an important role as a reducing agent (sulfate group) and as a hair protection agent (polyphenol group) [43,44]. In curly hair, disulfide bonds are connected, resulting in a wavy structure. The presence of SS in PIS shampoo induces breakage of disulfide bonds and loosening of curls, which straightens the hair. Conventional chemical-based hair-straightening methods can damage the hair structure, whereas structural damage is minimized when using the PIS shampoo, as the GTLE–DOPA complex coats the hair by binding to the residual thiol groups through the Michael addition reaction, thereby reinforcing the structure and smoothness of hair (Figure 1). The GTLE–DOPA complex also influences the surface properties of hair, leading to high hydrophobicity (for humidity protection), high charge dissipation (for an antistatic effect), and efficient ROS scavenging activity (for damage prevention), which promotes the features of healthy straightened hair.

### 3.2. Characterization of PIS Nanoparticles

**Figure 2:**
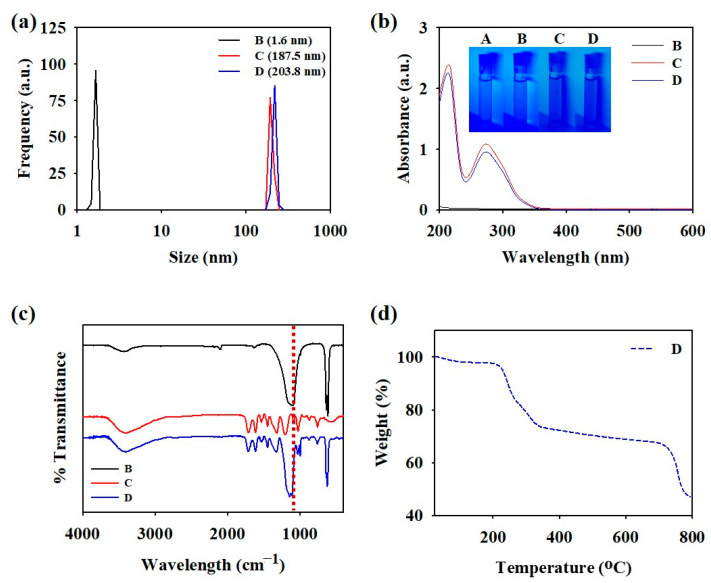
(**a**) Dynamic light scattering (DLS) measurement, (**b**) UV-Vis spectra, (**c**) FTIR spectra, and (**d**) thermal gravimetric analysis (TGA) measurement of PIS nanoparticles. A: DDW, B: sodium sulfate (SS), C: green tea leaf extract (GTLE)–DOPA, D: PIS mixture.

**Figure 3:**
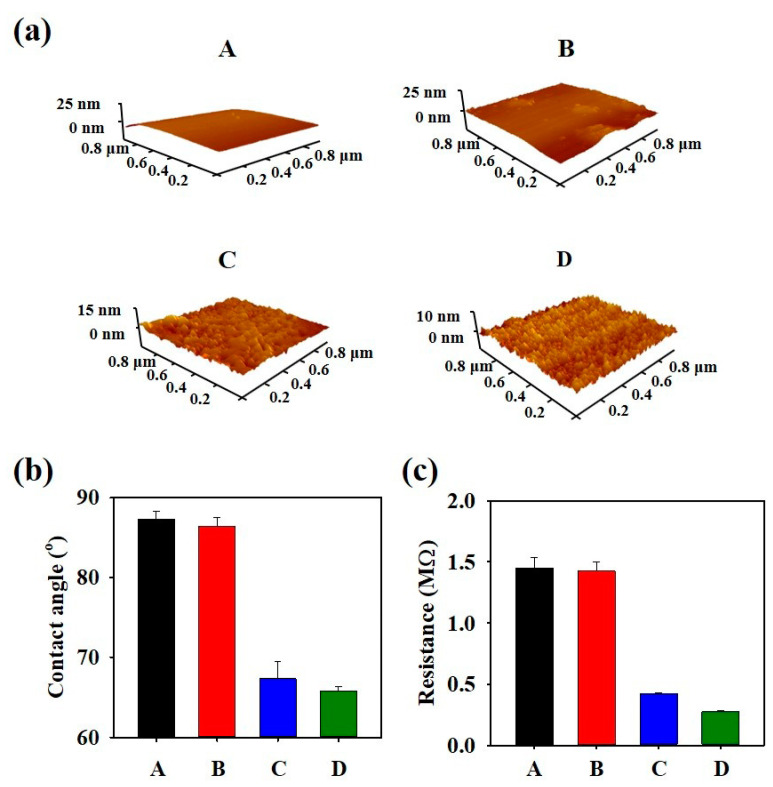
(**a**) Atomic force microscopy images, (**b**) water contact angle measurement, and (**c**) source meter resistance measurement of PIS-coated Si wafer. A: bare Si wafer, B: SS-coated Si wafer, C: GTLE–DOPA-coated Si wafer, D: PIS-coated Si wafer.

A mixture of GTLE–DOPA–SS nanoparticles (D, PIS) was characterized and compared with the controls (B, SS only and C, GTLE–DOPA only). The average size of the PIS nanoparticles was determined using dynamic light scattering (DLS) spectroscopy (Figure 2a). The diameter of the PIS nanoparticles (203.8 nm) was larger than those of the SS and GTLE–DOPA nanoparticles (1.60 and 187.5 nm, respectively). UV-Vis spectroscopy was performed to assess the optical and structural characteristics of the PIS nanoparticles (Figure 2b). The absorbance peaks at 225 and 280 nm corresponded to the π–π* of the GTLE–DOPA nanoparticles, which was attributed to the polyphenol aromatic group. The PIS nanoparticle structure was also confirmed using FTIR, which showed that the intense vibration peak at 1100 cm^−1^ corresponded to the asymmetric stretch of SO_4_, which was slightly shifted compared to that of sample B and was affected by the interaction between GTLE–DOPA and SS (Figure 2c). Moreover, TGA elucidated the degradation temperature of PIS nanoparticles at 230–330 °C and 730–770 °C, indicating high thermal stability (Figure 2d). The pH stability test showed no change in PIS nanoparticle pH after a long period of time, even after being mixed with shampoo (Figure S1a). PIS nanoparticles also showed structural stability in different pH (pH 7.4 and 5), indicated by unchanged UV-vis absorbance at 273 nm (Figure S1b,c). Furthermore, atomic force microscopy images showed that compared with the controls (A, B, and C), D possessed a rougher surface profile, indicating the hydrophobicity of the PIS nanoparticles (Figure 3a). Polyphenols have adhesive properties, allowing the PIS nanoparticles to easily coat the hair surface. To assess this feature, water contact angle measurements were conducted by coating the PIS nanoparticles onto the surface of a substrate (Si wafer). As shown in Figure 3b, samples C (23.2°) and D (15.3°) showed a significant change in contact angle compared with that of A (87.5°), owing to the GTLE–DOPA layer coating the substrate surface. In contrast, sample B (87.1°) showed a negligible change owing to its inability to attach to the surface, as it contained only SS. This difference in coatability resulted in differences in the electroconductivity of the substrate. As shown in Figure 3c, the resistance values of C (0.35 MΩ) and D (0.23 MΩ) were significantly lower than those of A (1.39 MΩ) and B (1.37 MΩ). This low resistance in D was achieved because of the presence of a polyphenol–sulfate layer that altered the conductivity of the substrate.

**Figure 4:**
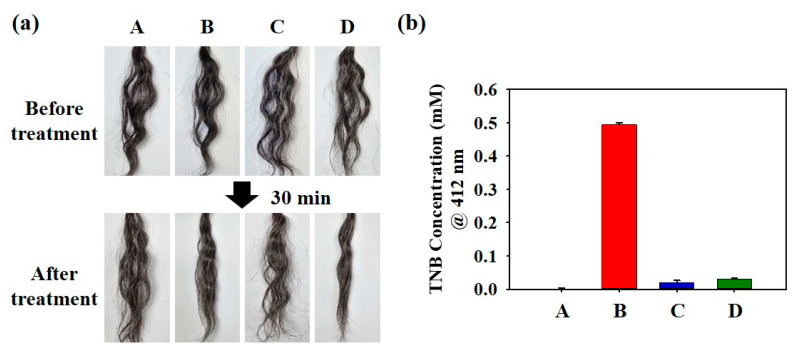
(**a**) Image of the actual hair-straightening effect of PIS nanoparticles and (**b**) Ellman’s assay using 5,5′-dithio-bis-(2-nitrobenzoic acid) (DTNB) to measure the amount of free thiol in the hair (indicated by TNB concentration quantified by absorbance measured at 412 nm). A: DDW, B: sodium sulfate (SS), C: green tea leaf extract (GTLE)–DOPA, D: PIS mixture.

Among the PIS components, SS was expected to produce hair-straightening effects, and the GTLE–DOPA mixture was expected to preserve the hair structure. To evaluate their performance, PIS nanoparticles were applied to curly hair samples, and their effect on the hair was assessed after 30 min. As shown in Figure 4a, samples B and D demonstrated a better straightening effect than samples A and C, owing to the presence of SS, which cleaved the disulfide bonds in the curly hair, thus loosening the structure and making the hair smooth. Next, Ellman’s assay was performed using DTNB to measure the level of free thiol in the hair (represented by the TNB concentration, quantified based on absorbance measured at 412 nm), which indicates the degree of hair damage after the straightening process (Figure 4b). Sample B contained a high concentration of TNB (0.494 mM), revealing abundant free thiol groups resulting from disulfide bond breakage. In contrast, although the curly hair was successfully straightened, the amount of free thiol in sample D (0.031 mM) was significantly lower than that in sample B because the polyphenols in the PIS nanoparticles bound with free thiol through the Michael addition reaction, thus reinforcing the hair structure. For samples A and C, the amount of free thiol was relatively low, owing to the unbroken disulfide bonds in the hair, as these samples did not contain SS.

**Figure 5:**
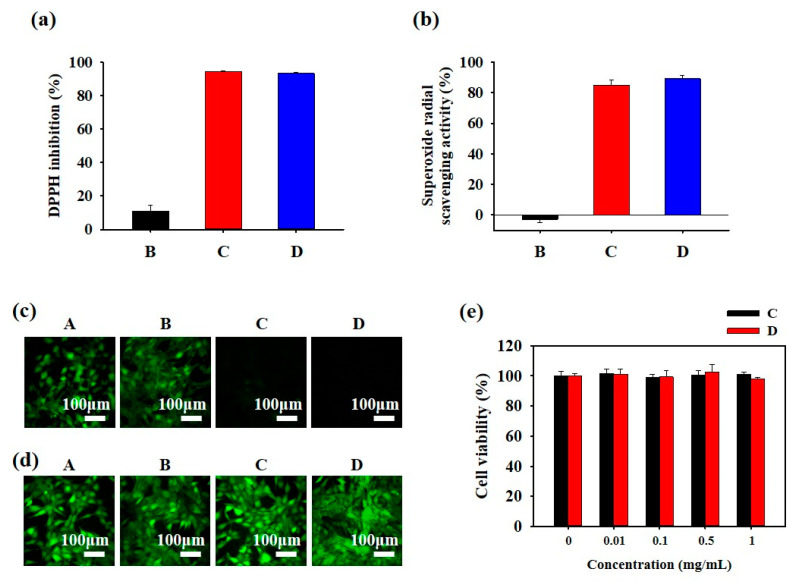
(**a**) 2,2-diphenyl-1-picrylhydrazyl (DPPH) assay, (**b**) superoxide dismutase (SOD) assay, (**c**) reactive oxygen species (ROS) cell staining assay, (**d**) live–dead staining assay, and (**e**) MTT assay of PIS nanoparticles. A: DDW, B: sodium sulfate (SS), C: green tea leaf extract (GTLE)–DOPA, D: PIS mixture.

As polyphenols possess antioxidant properties, PIS nanoparticles provide ROS scavenging activity to protect hair from potential damage. The ROS scavenging activity of PIS nanoparticles was evaluated using the DPPH and superoxide dismutase (SOD) assays. The DPPH assay revealed that samples C and D showed >90% DPPH inhibition owing to the presence of the GTLE–DOPA complex, whereas sample B showed notably low DPPH inhibition (approximately 10%) (Figure 5a). Furthermore, as shown in Figure 5b, samples C and D (>85% superoxide scavenging) were superior to sample B at scavenging superoxide radicals. Moreover, ROS scavenging activity was evaluated in hair cells using an ROS detection kit (Figure 5c). The results showed that the cells expressed almost no ROS when treated with samples C and D, as indicated by the absence of green fluorescence, in contrast to cells treated with samples A and B, which showed intense green fluorescence (high ROS). Cell viability of PIS nanoparticles was further evaluated using live and dead staining assays using CLSM (calcein AM = live/green, PI = dead/red) and an MTT assay. Confocal images confirmed that PIS demonstrated good biocompatibility, as indicated by intense green staining (Figure 5d). The MTT assay also confirmed almost 100% cell viability, indicating the biocompatibility of PIS and its safety for use on human hair (Figure 5e).

### 3.3. Evaluation of PIS Shampoo Performance for Straightening and Haircare

**Figure 6:**
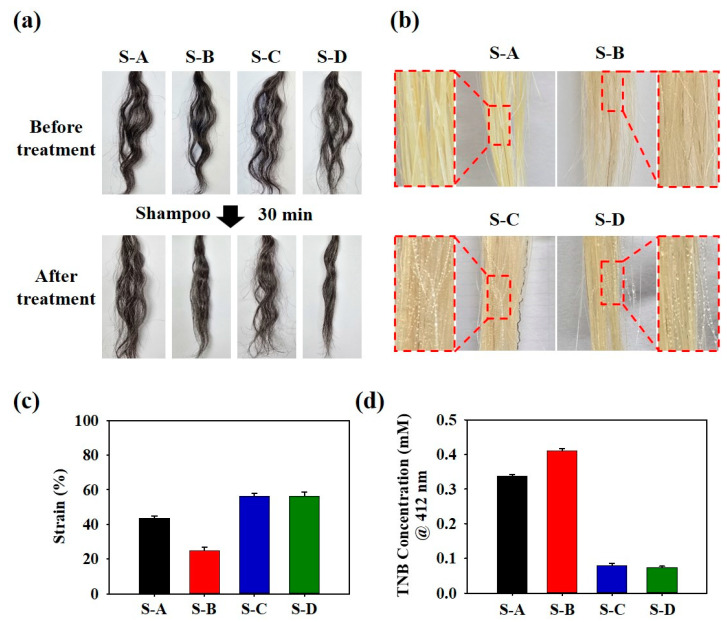
(**a**) Image showing the straightening effect on curly hair before and after treatment with PIS shampoo, (**b**) hydrophobicity test on the hair surface, (**c**) tensile strain test, and (**d**) Ellman’s assay of PIS shampoo-treated hair. S-A: only shampoo, S-B: shampoo + SS, S-C: shampoo + GTLE–DOPA, and S-D: PIS shampoo.

PIS nanoparticles were incorporated into a shampoo base to obtain PIS shampoo, which was tested on curly hair samples to determine its straightening performance. As shown in Figure 6a, the PIS shampoo (S-D) demonstrated an excellent straightening effect compared to that of the shampoo only (S-A), SS + shampoo (S-B), and GTLE–DOPA + shampoo (S-C). The better performance of S-D compared to that of S-B was achieved because of the structural reinforcement from GTLE–DOPA that binds with residual thiol groups following the reduction of disulfide bonds, thereby promoting a more stable and smoother straight form. The presence of polyphenols also provides hydrophobicity to the hair when coated on the hair surface, which is important for preventing water penetration under high-humidity conditions. Figure 6b shows that hair treated with S-C and S-D exhibited more hydrophobicity than that treated with S-A and S-B, owing to the polyphenol coating, as indicated by the distinct water droplet behavior on the hair surface. Polyphenols play an important role in enhancing the mechanical properties of hair after PIS shampoo application. The tensile strength tests were performed to ensure that the PIS shampoo treatment could straighten curly hair as well as strengthen the hair shaft to be more persistent towards hair loss. The results showed that hair treated with S-C and S-D possessed higher tensile strength (56.4 and 56.3% strain, respectively) than hair treated with S-A and S-B (43.6 and 24.8% strain, respectively) owing to the polyphenol coating (Figure 6c). Ellman’s assay was conducted to assess hair damage levels based on the amount of free thiol (Figure 6d). The results confirmed that the TNB concentration was significantly lower in the S-D-treated hair (0.059 mM) than in other samples (0.337, 0.410, and 0.079 for S-A, S-B, and S-C, respectively), indicating that the Michael addition reaction occurred between the polyphenol and free thiol. Ellman’s assay also revealed that S-D showed hair protection capability toward UV irradiation, indicated by low TNB concentration (0.088 mM) compared to the control (0.358 mM) (Figure S2).

**Figure 7:**
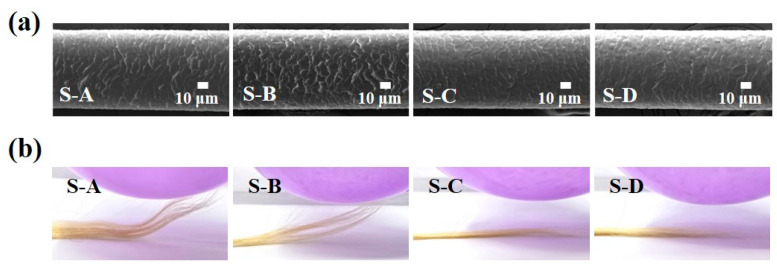
(**a**) Scanning electron microscopy (SEM) images and (**b**) antistatic test of PIS shampoo-treated hair. S-A: only shampoo, S-B: shampoo + SS, S-C: shampoo + GTLE–DOPA, and S-D: PIS shampoo.

The PIS shampoo was expected to smooth the hair cuticle owing to polyphenols that uniformly coat the hair. The SEM images show distinct smoothness after application of the shampoo (Figure 7a). In particular, hair treated with S-C and S-D exhibited smoother cuticles than those treated with S-A and S-B due to the polyphenol coating, thus confirming that polyphenol content is crucial for improving and restoring the hair cuticle. In addition, polyphenols in the PIS shampoo produced an antistatic effect by dissipating electrostatic charges. This function is required to prevent hair repulsion and entanglement owing to abundant electrostatic charges. The antistatic effect was observed in the rubber balloon test (Figure 7b). Hair treated with S-C and S-D demonstrated an antistatic effect, as the hair showed no attraction to the balloon. In contrast, hair treated with S-A and S-B adhered to the rubber balloon. This phenomenon confirmed that the electrostatic charge dissipation was promoted by π-π stacking, which increased surface conductivity. These data confirm that the PIS shampoo is capable of straightening curly hair as well as promoting hair integrity to achieve neat and healthy straight hair.

## 4. Conclusions

In this study, a PIS shampoo comprising polyphenols and inorganic sulfate was designed for multifunctional hair straightening, protection, and improvement. The combination of GTLE, DOPA, and SS promoted the hair-straightening effect by breaking disulfide bonds while preserving the structural integrity of the hair through the polyphenol–thiol Michael addition reaction. The polyphenol–thiol interaction was observed using Ellman’s assay, which showed a lower amount of free thiol in hair treated with PIS shampoo than in hair treated with the controls owing to the structural reinforcement from polyphenol coating on the hair shaft. The presence of polyphenols also improved the mechanical property (tensile strength) of PIS shampoo-treated hair, which is crucial to prevent hair breakage. The hydrophobicity of hair also improved, owing to the presence of the polyphenol coating on the hair surface, which is essential for protecting hair from a frizzy state, particularly under high-humidity conditions. Furthermore, the polyphenol content in the PIS shampoo facilitated smooth hair cuticles because of the polyphenol coating and generated an antistatic effect owing to high charge dissipation. In addition, the PIS shampoo exhibited antioxidant activity indicated by excellent ROS scavenging performance (>90%), and good biocompatibility with cells was observed. Thus, our PIS shampoo provides a novel approach for hair straightening that results in a desirable straight form while protecting the hair structure. We believe that the introduction of PIS shampoo in the haircare industry will be beneficial as it provides safe, simple usage and multiprotection for hair-straightening applications compared to conventional straightening approaches.

## Data Availability

The raw data supporting the conclusions of this article will be made available by the corresponding authors upon request.

## Supplementary Materials

The following supporting information can be downloaded at https://www.mdpi.com/article/10.3390/biomimetics10030132/s1: Supplementary Scheme S1. Chemical reaction for synthesizing PIS; Supplementary Figure S1. pH stability of PIS; Supplementary Figure S2. UV protection capability of PIS shampoo; Supplementary Table S1. Ratio between GTLE, DOPA, SS, and shampoo base to obtain PIS shampoo.
