# Supplementary material for: Polyphenol–Inorganic Sulfate Complex-Enriched Straightening Shampoo for Reinforcing and Restoring Reduced Hair Integrity
Source: Biomimetics (Basel). 2025 Feb 22;10(3):132. doi: 10.3390/biomimetics10030132 (PMC11939901; doi:10.3390/biomimetics10030132)
Supplement: Supplementary file 1 [file biomimetics-10-00132-s001.zip › biomimetics-3480717-supplementary.docx]

**Supporting Information**

**Polyphenol-Inorganic Sulfate Complex-enriched Straightening Shampoo for Reinforcing and Restoring Reduced Hair Integrity**

*Tae Min Kim^1^, Heung Jin Bae^1,2,^*, Sung Young Park^1,3,^**

^1^ Department of IT and Energy Convergence (BK21 FOUR), Korea National University of Transportation, Chungju 27469, Republic of Korea; ktm1120@a.ut.ac.kr

^2^ MODAMODA Corporation, Ltd., Songpa-gu, Seoul 05546, Republic of Korea

^3^ Department of Chemical and Biological Engineering, Korea National University of Transportation, Chungju 27469, Republic of Korea

***Corresponding authors:** Tel: +82-(0)43-841-5225, Fax: +82-(0)43-841-5220, E-mail: [newseowha@naver.com](mailto:newseowha@naver.com) (H.J.B), [parkchem@ut.ac.kr](mailto:parkchem@ut.ac.kr) (S.Y.P.)


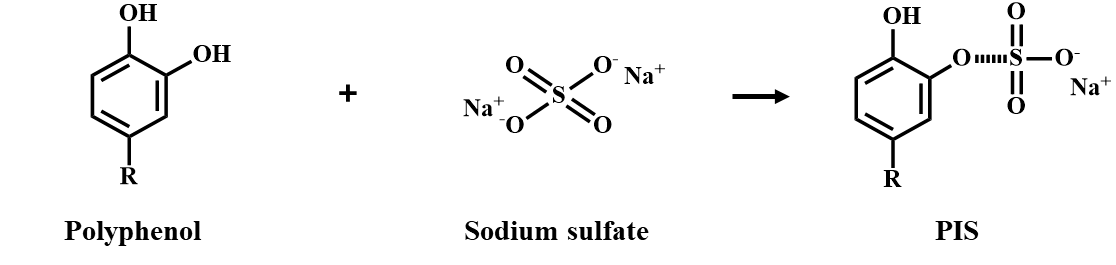


**Scheme S1.** Chemical reaction illustration for synthesizing PIS nanoparticle.

**
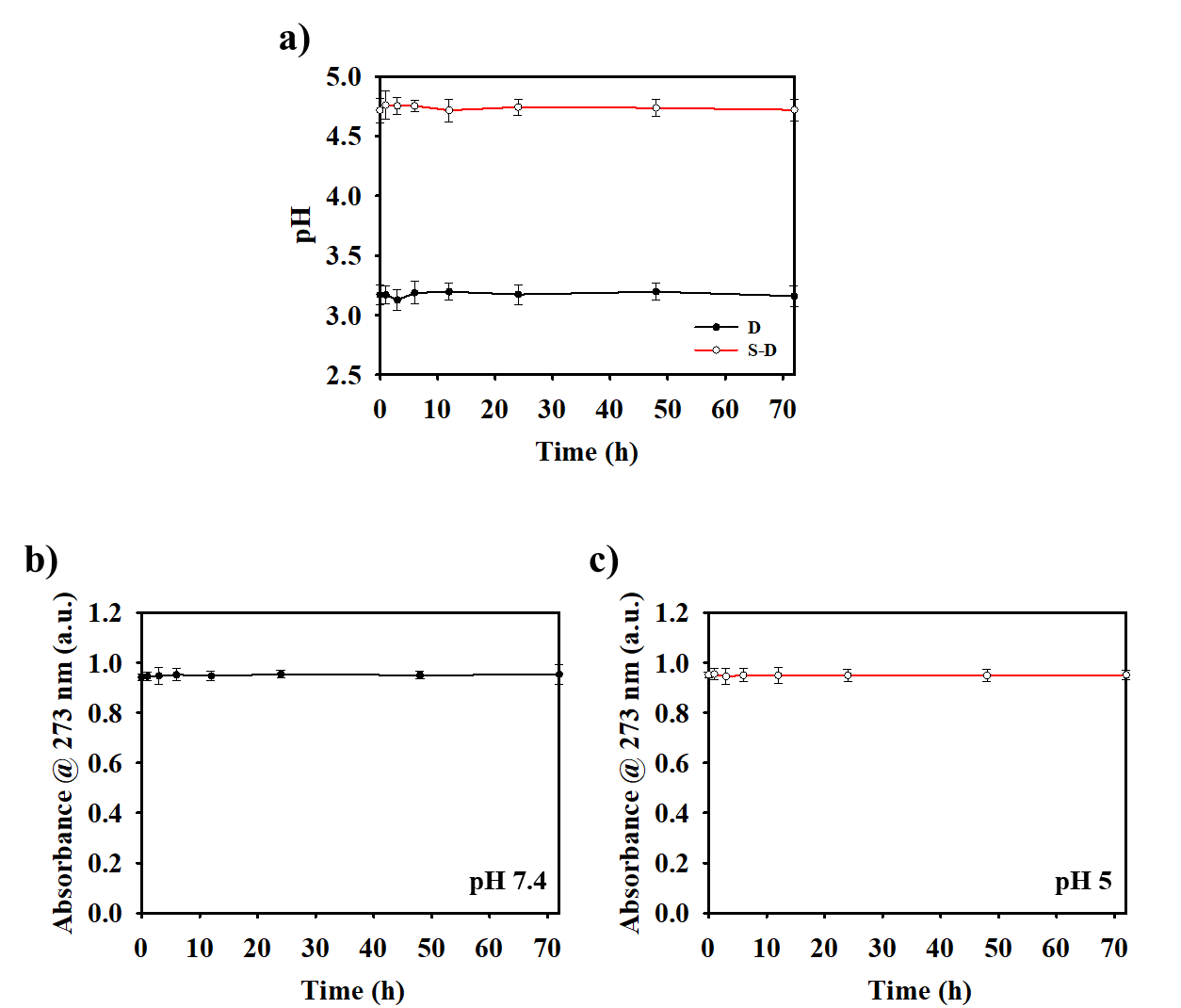
**

**Figure S1.** **(a)** pH stability of PIS nanoparticle (D) and PIS shampoo (S-D) after several periods. Effect of pH on PIS nanoparticle stability in **(b)** pH 7.4 and **(c)** pH 5.


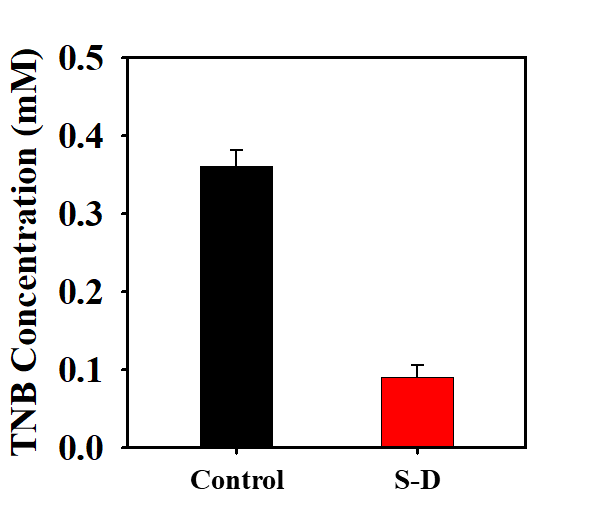


**Figure S2.** Ellman’s assay using 5,5’-dithio-bis-(2-nitrobenzoic acid) (DTNB) to indicate UV protection of PIS shampoo (S-D).

**Table S1.** Ratio between GTLE, DOPA, SS and shampoo base for obtaining PIS shampoo.

|  | **GTLE (%)** | **DOPA (%)** | **Sodium sulfate (%)** | **DDW (%)** | **Base shampoo (%)** |
| --- | --- | --- | --- | --- | --- |
| **A** | **0** | **0** | **0** | **100** | **0** |
| **B** | **0** | **0** | **13.66** | **86.34** | **0** |
| **C** | **4.73** | **0.63** | **0** | **94.64** | **0** |
| **D** | **4.12** | **0.55** | **13.03** | **82.30** | **0** |
| **S-A** | **0** | **0** | **0** | **9.09** | **90.91** |
| **S-B** | **0** | **0** | **1.41** | **8.96** | **89.63** |
| **S-C** | **0.45** | **0.06** | **0** | **9.04** | **90.45** |
| **S-D** | **0.45** | **0.06** | **1.41** | **8.92** | **89.16** |
